# Case Report: Chinese female patients with a heterozygous pathogenic *RPS6KA3* gene variant c.898C>T and distal 22q11.2 microdeletion

**DOI:** 10.3389/fgene.2022.900226

**Published:** 2022-08-15

**Authors:** Yan Cong, Hongxing Jin, Ke Wu, Hao Wang, Dong Wang

**Affiliations:** ^1^ Rehabilitation Department, Yiwu Maternity and Child Health Care Hospital, Yiwu, China; ^2^ Pediatric Department, Yiwu Maternity and Child Health Care Hospital, Yiwu, China; ^3^ Prenatal Diganosis Center, Yiwu Maternity and Child Health Care Hospital, Yiwu, China

**Keywords:** Coffin–Lowry syndrome, loss-of-function, intellectual disability, menstrual disorder, whole-exome sequencing, RPS6KA3 c.898C>T mutation

## Abstract

**Background:** Coffin–Lowry syndrome (CLS) [OMIM#303600] is a rare X-linked dominant syndrome. CLS is caused by highly heterogeneous loss-of-function mutations in the *RPS6KA3* gene (OMIM*300,075). CLS is characterized by intellectual disability (ID), short stature, tapered fingers, characteristic facial features, and progressive skeletal changes. Distal 22q11.2 microdeletion syndrome (OMIM#611867) is an autosomal dominant and recurrent genomic disorder. It mainly includes three types [distal type I (D–E/F), type II (E–F), and type III (F–G)] and exhibits variable clinical phenotypes (mild, moderate, or even normal): preterm birth, pre- and/or postnatal growth restriction, development delay, ID, behavioral problems, cardiovascular defects, skeletal anomalies, and dysmorphic facial features. We investigated the genetic etiology of a Chinese pedigree with ID, short stature, digit abnormalities, facial dysmorphism, and menstrual disorder. A heterozygous *RPS6KA3* gene variant c.898C>T (p.R300X) was identified in this familial case. Two female CLS patients with distal 22q11.2 microdeletion presented with more severe clinical phenotypes. We provided clinical characteristics of these Chinese female CLS patients.

**Case presentation:** We described a Chinese family with three affected females (the mother, the elder sister, and the proband). The mother and the elder sister had more severe clinical phenotypes (moderate facial dysmorphism, more severe cognitive impairment, and shorter stature). The common characteristic phenotypes are ID, short stature, facial dysmorphism, irregular menstruation, and cardiovascular disorders. Peripheral blood samples were collected from the pedigree. Whole-exome sequencing (WES) identified a heterozygous nonsense *RPS6KA3* gene variant c.898C>T (p.R300X). It was verified by Sanger sequencing. Copy number variation sequencing (CNV-seq) showed that both the mother and the elder sister carried a CNVseq [hg19] del (22) (q11.22-q11.23) (22997582–23637176)×0.5. RNA from peripheral blood samples was used for measuring the relative quantification of mRNA (expressed by exon 14 of *RPS6KA3*). The levels of mRNA relative expressions were significantly lower in the mother’s and the elder sister’s blood samples. The levels of mRNA relative expressions were significantly higher in the proband’s blood sample. X-chromosome inactivation (XCI) studies demonstrated that the proband showed extremely skewed XCI, and the XCI pattern of the elder sister was random.

**Conclusion:** Herein, we reported three Chinese female patients with a heterozygous nonsense *RPS6KA3* gene variant c.898C>T. Further genetic studies were performed. To our knowledge, Chinese patients with this variant have not been previously reported in the literature. The three female patients presented with variable degrees of severity. The clinical characteristics of these Chinese female CLS patients could expand the phenotypic spectrum of CLS. We helped physicians to understand the genotype–phenotype correlation further.

## Background

CLS is an X-linked dominant disorder characterized by ID, craniofacial features, and skeletal abnormalities. CLS is caused by heterozygous loss-of-function mutations in the *RPS6KA3* gene. A total of 70–80% of probands of CLS had no family history, and roughly 2/3 occurred *de novo*. A total of 20–30% of CLS patients had more than one affected family member (Pereira et al., 2010). With the use of WES, we reported a Chinese pedigree with a heterozygous pathogenic variant c.898C>T (p.R300X) in *RPS6KA3* gene. Further genetic studies were performed. A CNV was found in two female CLS patients with more severe clinical phenotypes, they may be associated with distal 22q11.2 microdeletion syndrome. We described clinical characteristics of these Chinese female CLS patients. Also, we hoped to improve the comprehensive understanding of CLS for pediatricians.

## Case presentation

**FIGURE 1:**
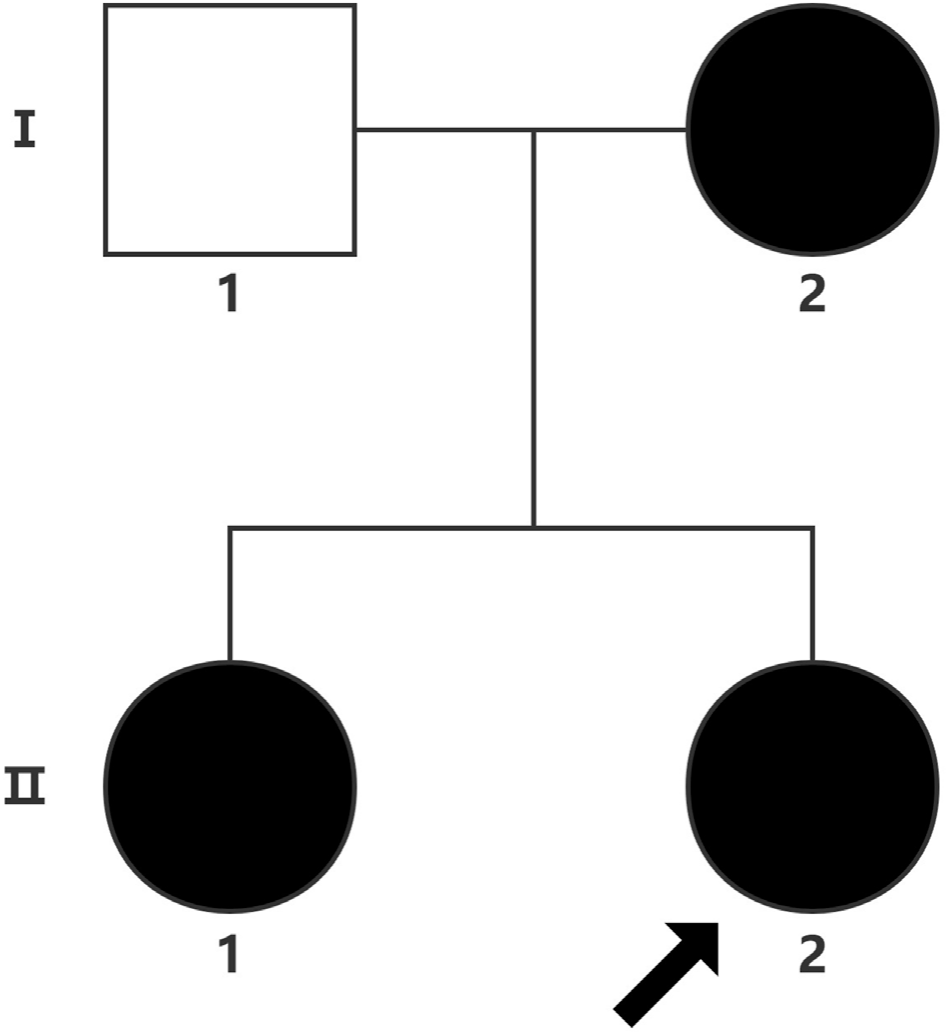
Proband (arrow).

**FIGURE 2:**
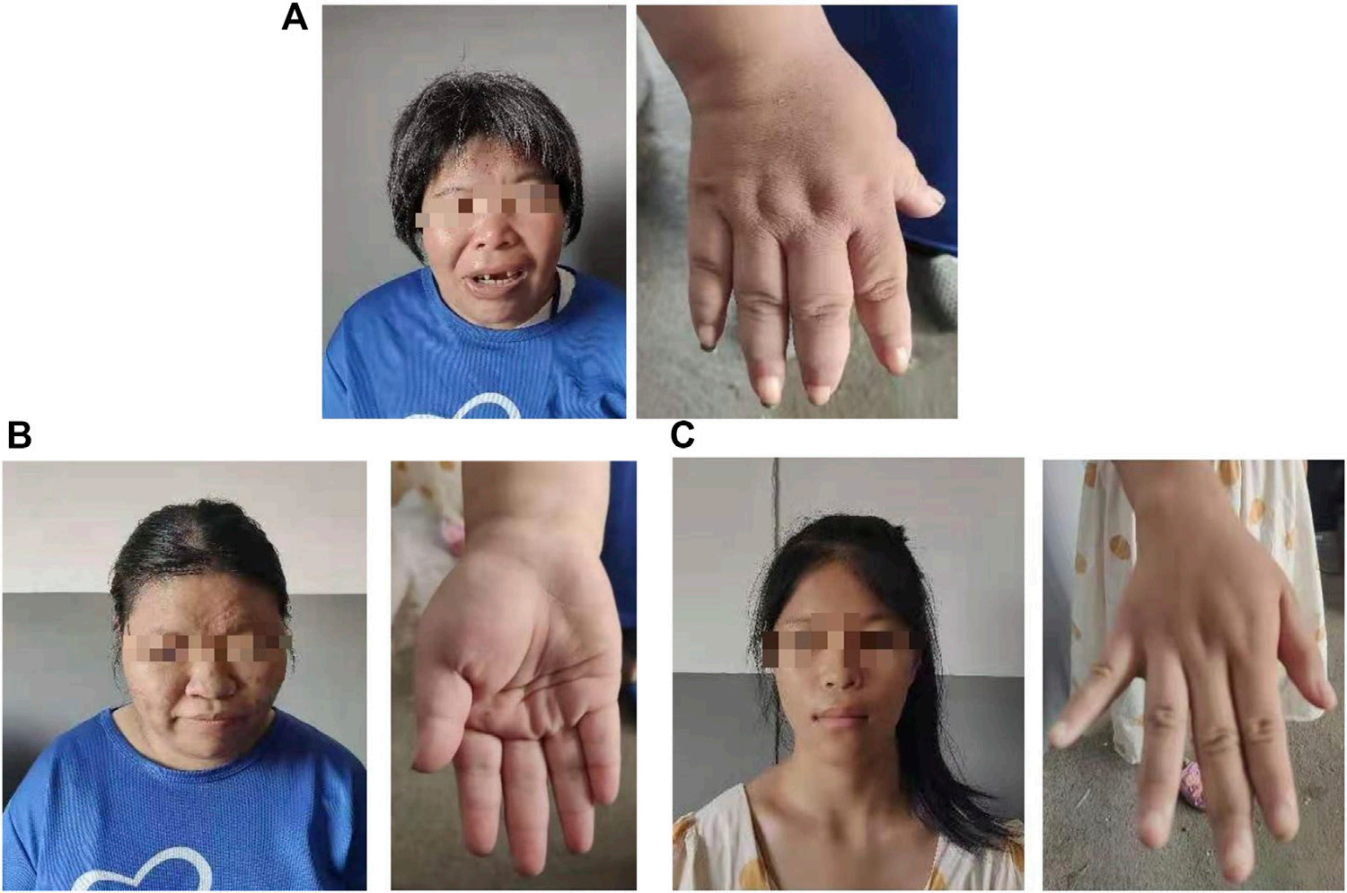
Hand and facial features of the mother **(A)**. Hand and facial features of the elder sister **(B)**. Hand and facial features of the proband **(C)**.

**FIGURE 3:**
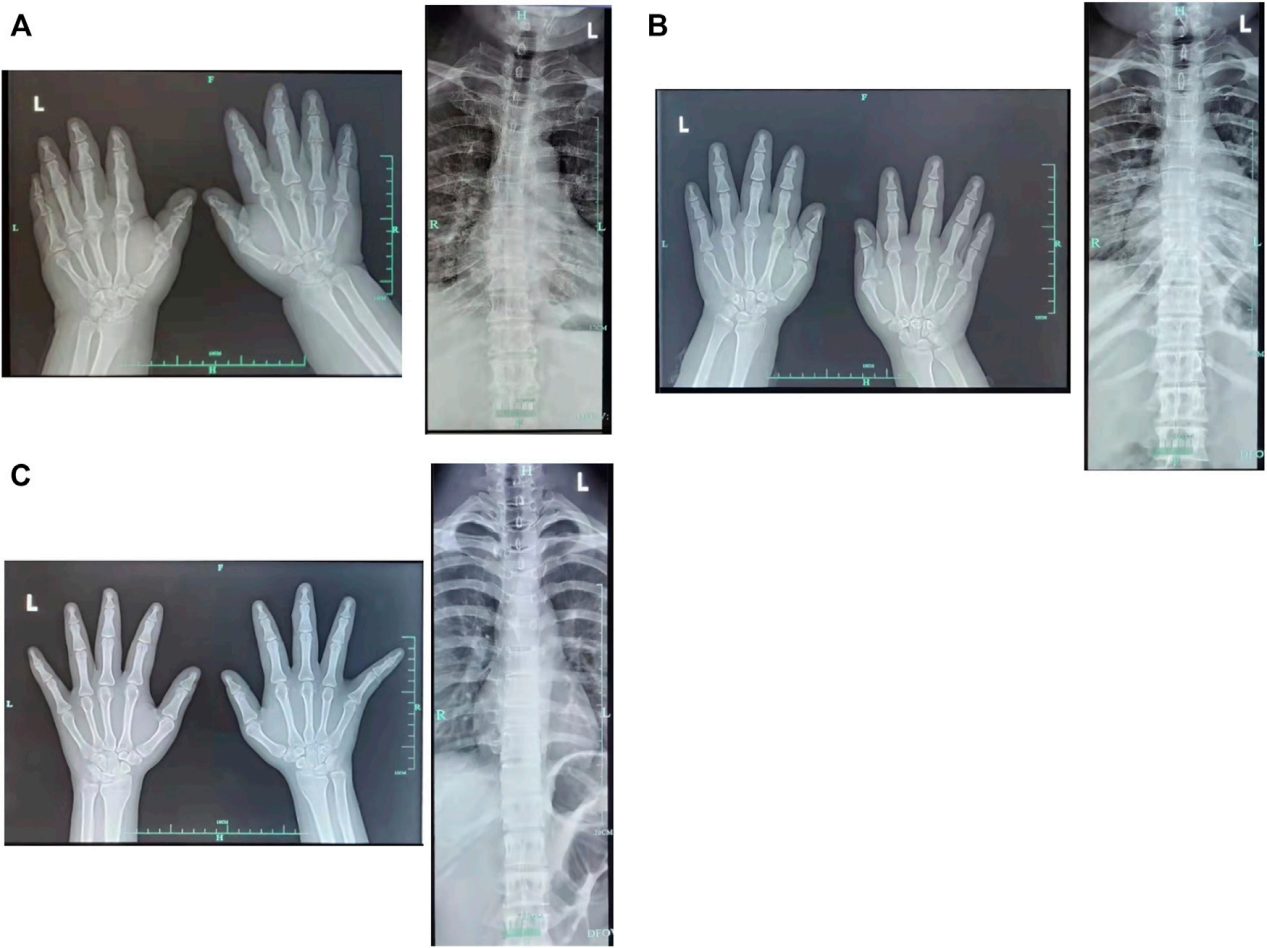
X-rays of hands and spines. **(A)** Mother **(B)** Elder sister **(C)** Proband.

The proband (a 17 year-old female patient) was found by us in the countryside. The proband had additional two affected family members (Figure 1). They had similar symptoms. The parents were residents of adjacent villages, and they did not have a consanguineous relation. The proband’s father was phenotypically normal. The proband and her elder sister (21 years old) were born at full-term by spontaneous vaginal delivery without complication in the perinatal and neonatal period. The weight and height of the proband were 56 kg and 161 cm, respectively. The weight and height of her elder sister were 72 kg and 142 cm, respectively. The weight and height of her mother (47 years old) were 65 kg and 140 cm, respectively. The cognitive impairment tests showed that MMSE scores of the proband, her elder sister, and mother were 13, 8, and 5, respectively. The proband had received education at a special school for 2 years. Now she can recognize some Chinese characters, and she has simple communication with people. She had a poor memory like always forgetting her own birthday. Her elder sister and mother could not recognize any Chinese characters. They had little communication with people. The three patients all had coarse craniofacial features including widely spaced downward-slanting palpebral fissures, low nasal bridge, blunt tip, broad nose, anteverted nares, and thick lips (Figure 2C). In addition, her elder sister had acne all over her face (Figure 2B), and her mother had a wide and open mouth, and thick lips with everted lower lip (Figure 2A). The elder sister’s and mother’s fingers tapered markedly from relatively wide proximally to narrow distally with small terminal phalanges and nails. The proband’s fingers did not show anything abnormal. During their adolescent period, they all had irregular menstruation. Echocardiography showed that they all had mild mitral and tricuspid valve regurgitation. X-rays of spines showed that only the mother had scoliosis (Figure 3A). X-rays of hands did not show any signs of abnormality.

## Neuropsychological tests

The mini-mental state examination (MMSE) (Pangman et al., 2000) is a 30-point questionnaire. MMSE is one of the most widely used brief screening instruments for measuring cognitive impairment. It has been modified and translated into a Chinese version. The optimal cut-off points were determined according to the education level. The optimal cut-off points were 16/17 for illiterate individuals, 19/20 for individuals with 1–6 years of education, and 23/24 for individuals with seven or more years of education (Li et al., 2016).

## Genetic tests

**FIGURE 4:**
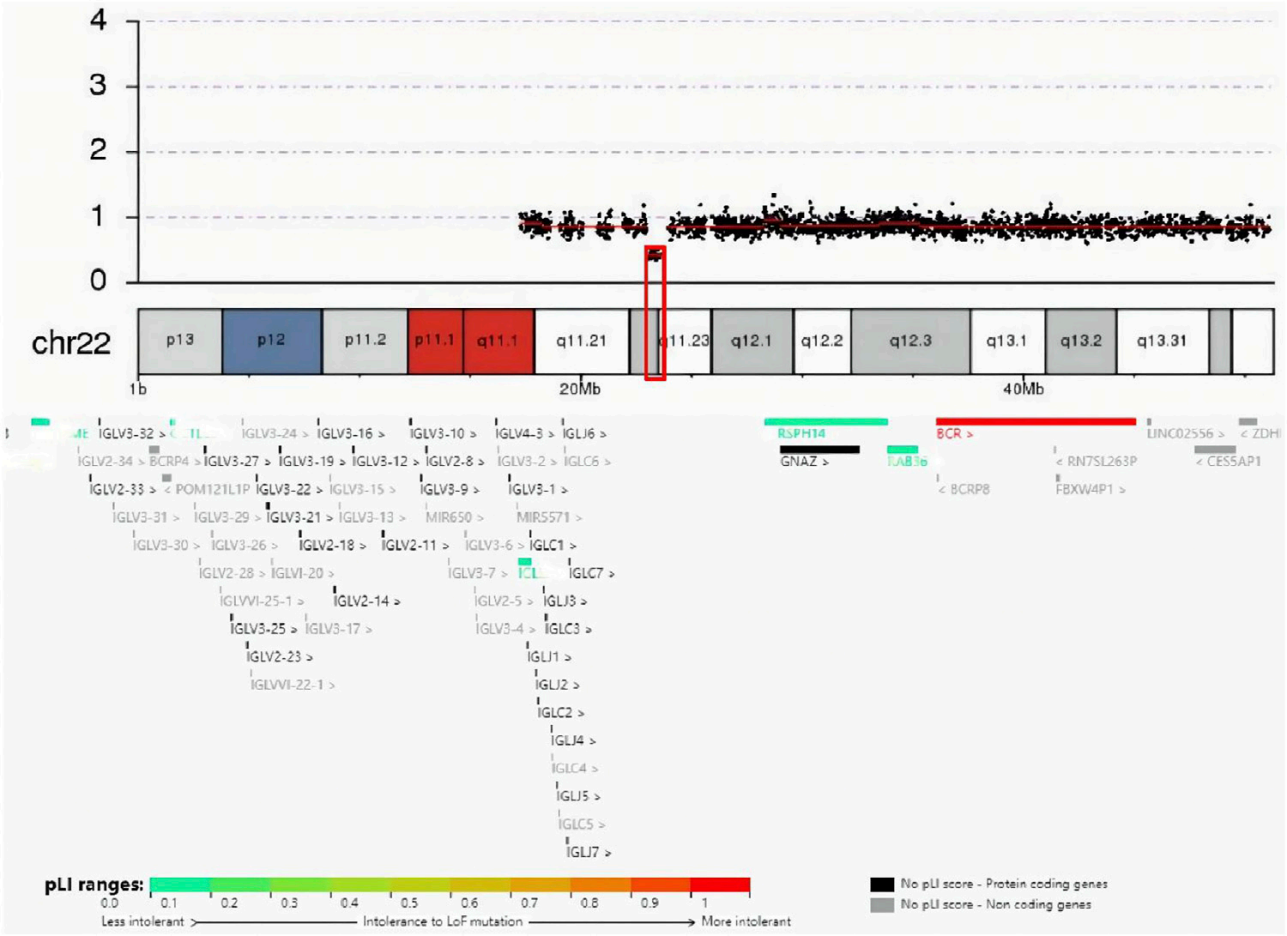
Result of CNV-seq was seq (hg19) del (22) (q11.22-q11.23) (22997582–23637176) × 0.5. In the deletion region (marked with a red box), genes were colored by the pLI score.

**FIGURE 5:**
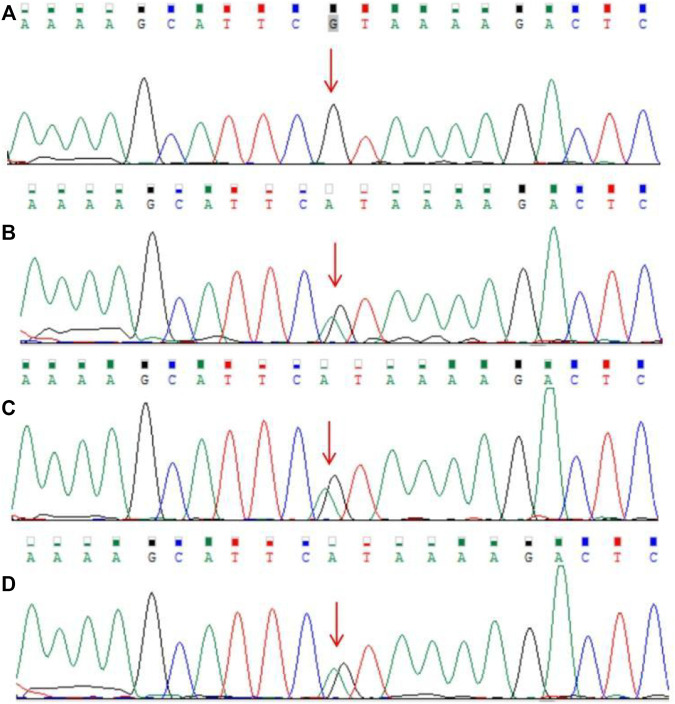
Results of Sanger sequencing. **(A)** Father (**B–D)**. Heterozygous *RPS6KA3* gene variant **(C)** 898C>T (NM_004,586.3) in the mother, the elder sister, and the proband (the variant was marked with a red arrow).

The guardian (the proband’s father) signed an informed consent for genetic analysis. Our legal ethics committee approved this genetic study. gDNAs were extracted from peripheral blood of the patients and phenotypically normal father for WES and CNVseq. Sanger sequencing was used for further verification. CNV-seq showed that both the mother and the elder sister carried a CNVseq [hg19] del (22) (q11.22-q11.23) (22997582–23637176) × 0.5 (Figure 4). WES identified a heterozygous nonsense *RPS6KA3* gene variant c.898C>T (p.R300X), which was verified by Sanger sequencing (Figure 5).

Levels of urine organic acids, plasma amino acids, lactate, and pyruvic acid were all normal in patients. Conventional G-banded chromosome analysis all showed a 46, XX karyotype. The results of genetic metabolic disease screening were all negative in patients.

**FIGURE 6:**
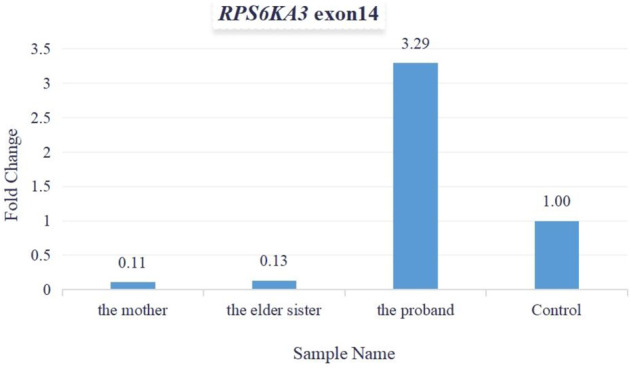
Fold change was used for calculating mRNA relative expression. The levels of mRNA relative expression were significantly lower in the mother’s and the elder sister’s blood samples, and the levels of mRNA relative expression were significantly higher in the proband’s blood sample.

As per the guidelines of the American College of Medical Genetics and Genomics (ACMG) for interpreting sequence variants (Richards et al., 2015), this variant was pathogenic (PVS1+PM2+PP1+PP3+PP4+PP5). Total cellular RNA was isolated from patients’ peripheral blood for measuring the relative quantification of mRNA (expressed by exon 14 of the *RPS6KA3* gene). The quantitative PCR (qPCR) data demonstrated that the levels of mRNA relative expression were significantly lower in the mother’s and the elder sister’s blood samples, and the levels of the mRNA relative expression were significantly higher in the proband’s blood sample (Figure 6).

gDNAs from peripheral blood samples of the two sisters and their mother were used for XCI analysis. The XCI studies demonstrated that the proband showed extremely skewed XCI and the XCI pattern of the elder sister was random.

## Discussion

CLS is a well-described and rare X-linked dominant syndrome. CLS is caused by highly heterogeneous loss-of-function mutations in the *RPS6KA3* gene. The ribosomal protein *S6 kinase A3* (*RPS6KA3*) gene is located in Xp22.12, and it encodes ribosomal protein S6 kinase alpha-3 (UniProtKB-P51812). This transcript of the *RPS6KA3* gene has 22 coding exons, a transcript length of 7987 base pairs, and a translation length of 740 amino acids (NM_004586.3). The RPS6KA3 is one of the serine/threonine-protein kinase. RPS6KA3 acts as one of many downstream effectors of the MAP-kinase ERK in the RAS-RAF-MEK-ERK signaling pathway. RPS6KA3 plays an important role in cell-cycle progression, differentiation, and cell survival (Fischer and Raabe, 2018). RPS6KA3-deficient mice displayed spatial learning impairment (Poirier et al., 2007). In *RPS6KA3* knock-out mice, proliferation of adult-generated neurons was decreased and no pro-survival effect of learning was observed. Castillon et al. (2018) suggested that decreased young newborn neurons were associated with deficient long-term memory recall. Likewise, poor memory and learning impairment have been observed in our CLS patients.

Based on the authors’ experience, the prevalence of CLS was 1:40,000–1:100,000 (Pereira et al., 2010). CLS is usually characterized by severe-to-profound ID, characteristic craniofacial features, tapered fingers, and musculoskeletal features. Carrier females are less severely impaired. Approximately 14% of affected males and 5% of affected females had cardiovascular diseases (Hanauer and Young, 2002). A total of 13–20% of male CLS and 3–7% of female CLS presented with stimulus-induced drop episodes (SIDEs) (Rojnueangnit et al., 2014). In addition, a few CLS patients presented with diabetes type 2 (Boulos et al., 2021), epileptic seizures (Gschwind et al., 2015), growth retardation (Lv et al., 2019), and hearing or vision problems (Hunter, 2002). The risk of childhood-onset schizophrenia (COS) might be increased in CLS patients (Ambalavanan et al., 2019). Neuroimaging studies demonstrated some brain abnormalities, such as thinning and agenesis of the corpus callosum, mild dilatation of the ventricles, reduced gray- and white-matter volumes, and impacted cerebellum and hippocampus volumes (Wang et al., 2006; Kesler et al., 2007).

In this familial case, these three patients all had the ability to walk, no loss of hearing or vision. For now, there are no signs of SIDEs and epileptic seizures in them. They all had irregular menstruation at puberty. The length of their menstrual cycles kept changing, such as 45 days, 60 days, and 180 days. This symptom has not been previously reported in the literature. Ultrasonography of ovaries and uteruses all showed normal results. They refused to take brain magnetic resonance imaging (MRI) scans.

**FIGURE 7:**
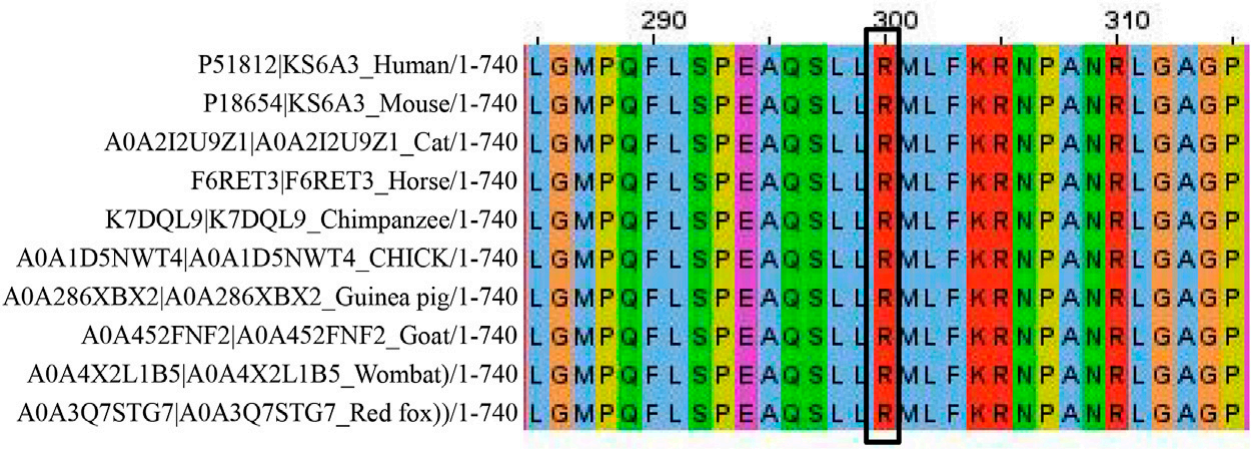
In total, 30 amino acids surrounded the variant position (marked with a black box).

According to the ACMG standards and guidelines for the interpretation of sequence variants, we interpreted c.898C>T (p.R300X) as a pathogenic variant. This variant caused a premature termination of codon, resulting in strong protein truncation (more than 50% of protein length was missing) (PVS1). It was absent from normal population databases (Exome Sequencing Project, 1000 Genomes Project, the Genome Aggregation Database, and Exome Aggregation Consortium) (PM2). Silico predictive algorithms (SIFT, PolyPhen-2, and Mutation Taster) of pathogenicity all showed that this variant was damaging. This variant was segregated from the disease in multiple family members (PP1). The analysis of conserved sequences suggested that this variant was located in highly conserved sequences across several species (Figure 7) (PP3). Three female patients’ phenotypes and family histories were highly specific for CLS (PP4). Tos et al. (2015) reported that a familial CLS case was caused by the same variant (PP5).

As shown in Table 1, the mother and the elder sister had the similar symptoms. They were more severely impaired than the proband. They presented with shorter stature, coarser facial features, tapered fingers, and poorer cognition. We speculated that more severe phenotypes may be connected with the CNV [ del (22) (q11.22-q11.23) (22997582–23637176)]. According to a proposed categorization system (Mikhail et al., 2014), they might be involved with distal 22q11.2 microdeletion syndrome. A cluster of low-copy repeats (LCRs) from A to H in chromosome 22q11.2 (chr22:17,900,001–25,900,000) mediated nonallelic homologous recombination and 22q11.2 chromosomal rearrangements. In the light of a systematic clinical overview of ClinGen curation (Xue et al., 2021), this CNV interval region contained the distal type II (E–F) region (chr22:23,119,414–23,649,111), and overlapped with the distal type I (D–E/F) region (chr22:21,917,117–23,649,111). Deletion of distal type I (D–E/F) or type II (E–F) regions may exhibit variable clinical phenotypes (mild, moderate, or even normal): prenatal growth restriction, short stature, ID, language delay, dysmorphic facial features, skeletal anomalies, cardiovascular defects, behavior problems, genitourinary anomalies, feeding problems, hypotonia, immune deficiency, and hypocalcemia (Burnside, 2015). Taken together, it was tempting to speculate that the CNV in the mother and elder sister made their phenotypes worse than the proband.

**TABLE 1:** Summary of genotype and phenotypes of the three female patients.

Genotype	Proband	Elder sister	Mother
*RPS6KA3* variant (NM_004,586.3)	c.898C>T	c.898C>T	c.898C>T
CNV	-	del (22) (q11.22-q11.23) involved with distal 22q11.2 microdeletion syndrome	del (22) (q11.22-q11.23) involved with distal 22q11.2 microdeletion syndrome
XCI pattern	Extremely skewed	Random	ND
mRNA relative expression (*RPS6KA3* exon 14)	Significantly higher	Significantly lower	Significantly lower
**Phenotypes of CLS (have not been reported)**			
Irregular menstruation	+	+	+
**Phenotypes of CLS (previously reported)**			
Cognitive impairment	Simple communication, little literacy, poor memory, ID	No communication or literacy, poor memory, ID	No communication or literacy, poor memory, ID
Dysmorphic facial features	Mild	Moderate	Moderate
Stature	Normal (161 cm)	Short stature (142 cm)	Short stature (140 cm)
Fingers	-	Markedly tapered	Markedly tapered
Skeletal anomalies	-	-	Scoliosis
SIDEs	-	-	-
Cardiovascular diseases	Mild mitral and tricuspid valve regurgitation	Mild mitral and tricuspid valve regurgitation	Mild mitral and tricuspid valve regurgitation
Diabetes	-	-	-
Seizures	-	-	-
Hearing or vision problems	-	-	-
Growth retardation	-	+	ND

−: negative; **+**: positive; XCI, X-chromosome inactivation; ND; no data; SIDEs, stimulus-induced drop episodes; ID, intellectual disability.

The two sisters showed different XCI patterns. We did not find a correlation between XCI patterns and phenotypic severity. It should be noted that the XCI pattern might be different in different tissues. Thus, XCI analysis in lymphocytes did not rule out this possibility. Also, the connection between the levels of mRNA (expressed by mutant *RPS6KA3* in blood samples) and phenotypic severity should be studied further. As we know, mRNA of the *RPS6KA3* gene is highly expressed in skeletal muscle. But we did not perform muscle biopsies, as we thought that it was kind of hard to get some tissues such as skeletal muscle, skin, or brain.

To sum up, severity of clinical features may be markedly variable. If possible, getting some tissues such as skeletal muscle or skin may be more useful for further genetic studies. These three female patients exhibited variable degrees of severity. Clinical characteristics of our CLS patients expanded the phenotypic spectrum of CLS. We hoped that our phenotypic and genetic studies could improve the comprehensive understanding of CLS for pediatricians.

## Data Availability

The datasets for this article are not publicly available due to concerns regarding participant/patient anonymity. Requests to access the datasets should be directed to the corresponding author.

## Abbreviations

CLS, Coffin–Lowry syndrome; RPS6KA3, ribosomal protein S6 kinase A3; gDNA, genomic DNA; Trio-WES, trio-based whole-exome sequencing; NV, copy number variation; MMSE, the mini-mental state examination; MRI, magnetic resonance imaging; SIDAs, stimulus-induced drop attacks; XCI, X-chromosome inactivation; COS, childhood-onset schizophrenia (COS).
