# Supplementary figures and images for: Case Report: Chinese female patients with a heterozygous pathogenic RPS6KA3 gene variant c.898C>T and distal 22q11.2 microdeletion
Source: Front Genet. 2022 Aug 15;13:900226. doi: 10.3389/fgene.2022.900226 (PMC9420874; doi:10.3389/fgene.2022.900226)

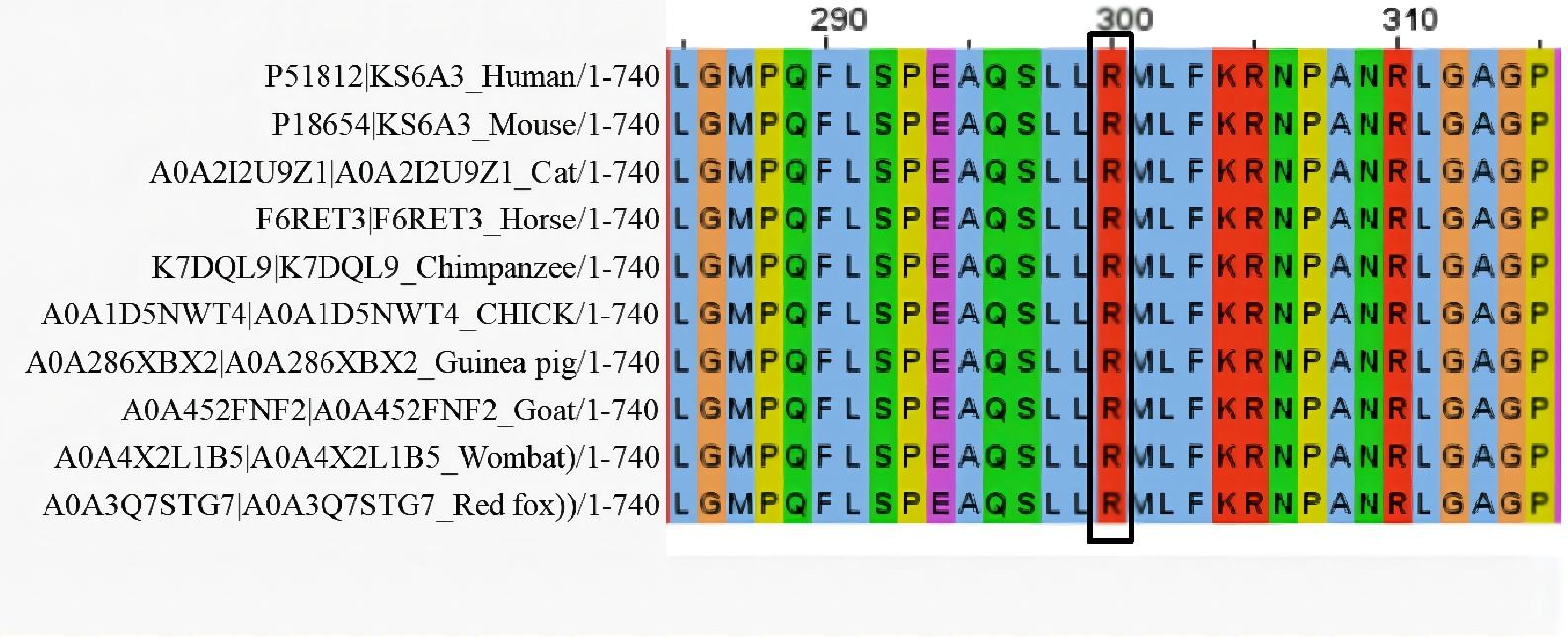

Supplement: Supplementary file 1 [file Image3.JPEG]

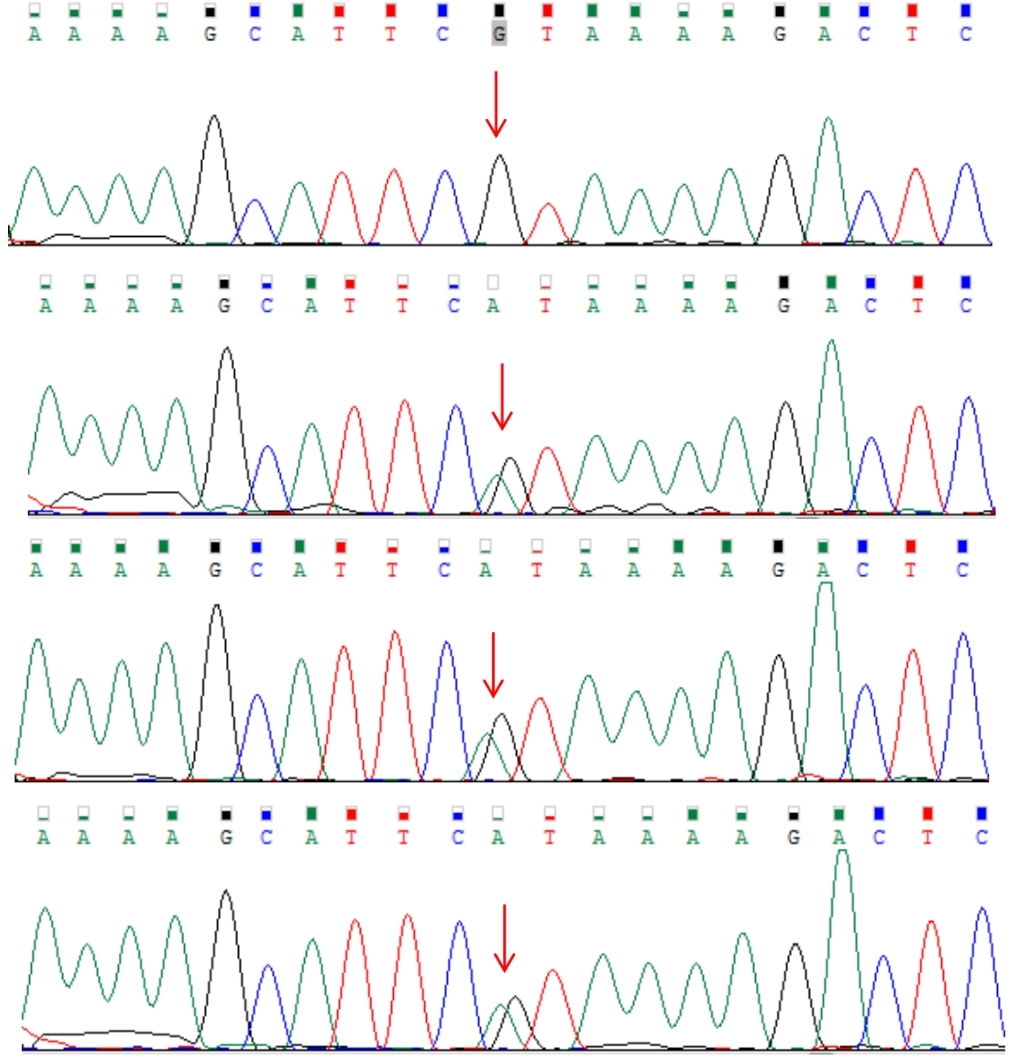

Supplement: Supplementary file 2 [file Image1.JPEG]

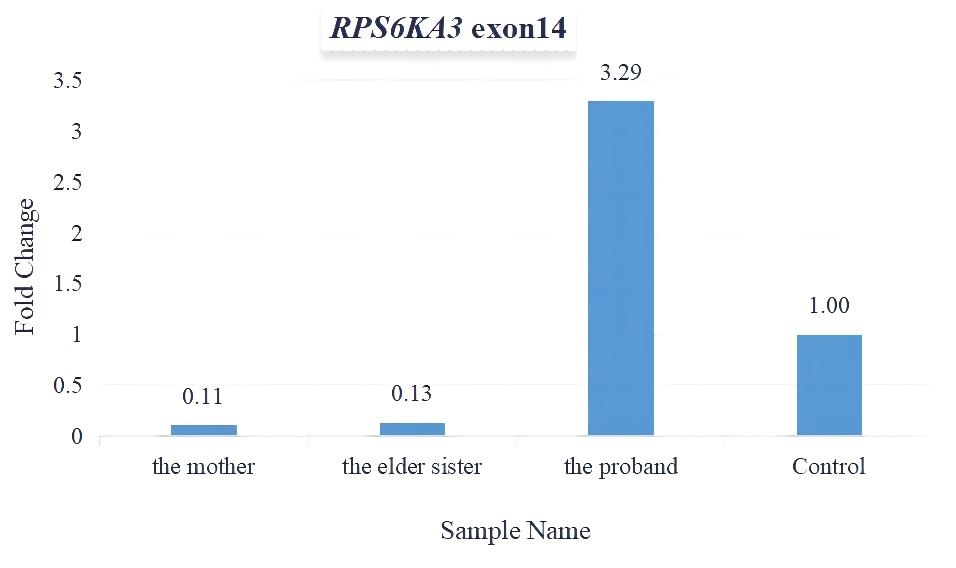

Supplement: Supplementary file 3 [file Image2.JPEG]
